# Insertion of LINE-1 Retrotransposon Inducing Exon Inversion Causes a Rotor Syndrome Phenotype

**DOI:** 10.3389/fgene.2019.01399

**Published:** 2020-01-31

**Authors:** Donghu Zhou, Saiping Qi, Wei Zhang, Lina Wu, Anjian Xu, Xiaojin Li, Bei Zhang, Yanmeng Li, Siyu Jia, Hejing Wang, Jidong Jia, Xiaojuan Ou, Jian Huang, Hong You

**Affiliations:** ^1^Experimental Center, Beijing Friendship Hospital, Capital Medical University, Beijing, China; ^2^Clinical Research Center for Rare Liver Diseases, Capital Medical University, Beijing, China; ^3^National Clinical Research Center for Digestive Diseases, Beijing Friendship Hospital, Beijing, China; ^4^Liver Research Center, Beijing Friendship Hospital, Capital Medical University, Beijing, China; ^5^Beijing Key Laboratory of Translational Medicine on Liver Cirrhosis, Beijing Friendship Hospital, Beijing, China

**Keywords:** Rotor syndrome, *SLCO1B1*, *SLCO1B3*, mutation, LINE-1 retrotransposon

## Abstract

Rotor syndrome, a rare autosomal-recessive genetic disorder characterized by conjugated hyperbilirubinemia, is caused by biallelic pathogenic variants in both *SLCO1B1* and *SLCO1B3* genes. Long interspersed nuclear elements (LINEs) make up about 17% of the human genome and insertion of LINE-1 in genes can result in genetic diseases. In the current study, we examined *SLCO1B1* and *SLCO1B3* genes in two Chinese patients diagnosed with Rotor syndrome based on laboratory tests. In one patient, a novel exon 4 inversion variant was identified. This variant may have been induced by LINE-1 retrotransposon insertion into *SLCO1B3* intron 3, and was identified using genome walking. Splicing assay results indicated that the exon inversion, resulting in *SLCO1B3* exon 4 (122 bp) exclusion in the mature mRNA, might generate a premature termination codon. Here, we describe an exon inversion contributing to the molecular etiology of Rotor syndrome. Our results may inform future diagnoses and guide drug prescriptions and genetic counseling.

## Introduction

Rotor syndrome is a benign condition that presents with conjugated hyperbilirubinemia (Jirsa et al., 1993) and is inherited in an autosomal-recessive manner. The prevalence of Rotor syndrome is unknown but is estimated to be quite low (<1:1,000,000). It is unknown if the prevalence of Rotor syndrome varies among different populations. Rotor syndrome diagnosis is established when the patient presents with isolated, predominantly conjugated hyperbilirubinemia without other liver injury and with typical cholescintigraphy findings. The clinical features of Rotor syndrome usually begin shortly after birth or in childhood. Although no specific treatment is required for Rotor syndrome, it needs to be clearly differentiated from other inherited bilirubin clearance disorders that can present with either conjugated or unconjugated hyperbilirubinemia, including Gilbert syndrome, Dubin-Johnson syndrome, and Crigler-Najjar syndrome (types I and II). Thus, the genetic detection of pathogenic variants is indispensable for distinguishing Rotor syndrome from other hereditary metabolic diseases, especially Dubin-Johnson syndrome, if no typical histopathological evidence is available.

Rotor syndrome is caused by homozygous or compound heterozygous pathogenic variants in both *SLCO1B1* and *SLCO1B3* genes. Such pathogenic variants result in complete functional deficiency of OATP1B1 and OATP1B3 proteins (van de Steeg et al., 2012) and inhibition of conjugated bilirubin uptake into the liver. At least one wild-type *SLCO1B1* or *SLCO1B3* allele prevents Rotor syndrome. Only a few studies have reported co-occurring pathogenic variants in *SLCO1B1* and *SLCO1B3* in patients from Japan, the Philippines, and Saudi Arabia and in patients of mixed central European descent (van de Steeg et al., 2012; Kagawa et al., 2015).

In human genome, LINE-1 is the only autonomous active transposable element, and comprise ~17% of the draft sequence (Lander et al., 2001). Insertion of the LINE-1 into gene loci can cause various forms of genetic instability, including extra nucleotide insertions, exon deletions, or chromosomal inversion (Symer et al., 2002; Wheelan et al., 2005; Zemojtel et al., 2007; Cordaux and Batzer, 2009; Adney et al., 2019). Here, we report two patients with Rotor syndrome in whom several pathogenic *SLCO1B1* and *SLCO1B3* variants were identified. Moreover, we identified a LINE-1-induced inversion variant through genome walking analysis.

## Materials and Methods

### Patients

Two patients with Rotor syndrome (patient R1, male, 15 years old and patient R2, male, 26 years old) were recruited from the China Registry for Genetics/Metabolic Liver Disease. Both patients underwent a standard biochemical and liver histopathology examination (Jirsa et al., 1993), and displayed conjugated hyperbilirubinemia (patient R1: total bilirubin (TBil) 103 μmol/L, direct bilirubin (DBil) 59.7 μmol/L; patient R2: TBil 101 μmol/L, DBil 96 μmol/L). Their livers were histologically normal. The patients both provided written informed consent, and this study was approved by the Ethics Committee of Beijing Friendship Hospital, Capital Medical University.

### Identification of *SLCO1B1* and *SLCO1B3* Gene Variants

Genomic DNA was extracted using the Blood Genomic DNA Kit (CoWin Biosciences, Beijing, China) following the manufacturer’s instructions. All *SLCO1B1* and *SLCO1B3* exons, and at least 50 bp of flanking sequence, were amplified using primers listed in **Table S1**. PCR amplification was performed in a PCR cycler (ProFlex; Applied Biosystems, CA, USA). The products were sequenced in the forward and reverse orientations by Tianyi Huiyuan Bioscience and Technology Inc. (Beijing, China).

The obtained sequences were aligned to the relevant reference sequences (*SLCO1B1*, NG_011745.1 or *SLCO1B3*, NG_032071.1) and variants were detected using Mutation Surveyor 5.01 software (Dong and Yu, 2011; Minton et al., 2011). The clinical and predicted pathogenicity of the variants were determined using VarCards (http://varcards.biols.ac.cn/) (Li et al., 2018).

### Genome Walking and Splice Site Analysis

A genome walking strategy was used to identify the unknown sequence between *SLCO1B3* exons 3 and 5 in patient R2. The Genome Walking Kit (TaKaRa Bio, Shiga, Japan) was used according to the manufacturer's instructions. The random primers used (AP primers) were purchased from TaKaRa, and the sequences of the 12 specific primers (SP primers) used are listed in **Table S1**. The amplification products obtained were separated by agarose gel electrophoresis and extracted from the gel. The products were inserted into a simple T-vector for sequencing. Splice site analysis was performed using Human Splicing Finder online software (http://www.umd.be/HSF/).

### Construction of Splicing-Reporter Vector (pET-01) and Splicing Assay

The pET-01 vector was used for both exon-trapping and as a splicing reporter. The wild-type or inverted exon 4 and the flanking intronic sequences (250 bp) were cloned into the pET-01 vector using BamHI and NotI restriction enzyme sites. The plasmids were transfected into cultured 293T and Huh-7 cells (Lipofectamine LTX Reagent, Thermo Fisher Scientific, CA, USA). After 24 h, the cells were harvested and total RNA was isolated using TRIzol RNA isolation reagents (Invitrogen, CA, USA). RNA was reverse-transcribed to cDNA using a cDNA synthesis kit (Transcriptor First Strand cDNA Synthesis Kit, Roche Diagnostic Systems, NJ, USA) following the manufacturer's instructions. The primers were designed to amplify the splicing products of the pET-01 vector (**Table S1**). After PCR amplification, agarose gel electrophoresis was used to determine amplicon length and to deduce whether exon 4 was present in the mature mRNA.

## Results

### Mutation Spectrum of the Patients With Rotor Syndrome

**Figure 1:**
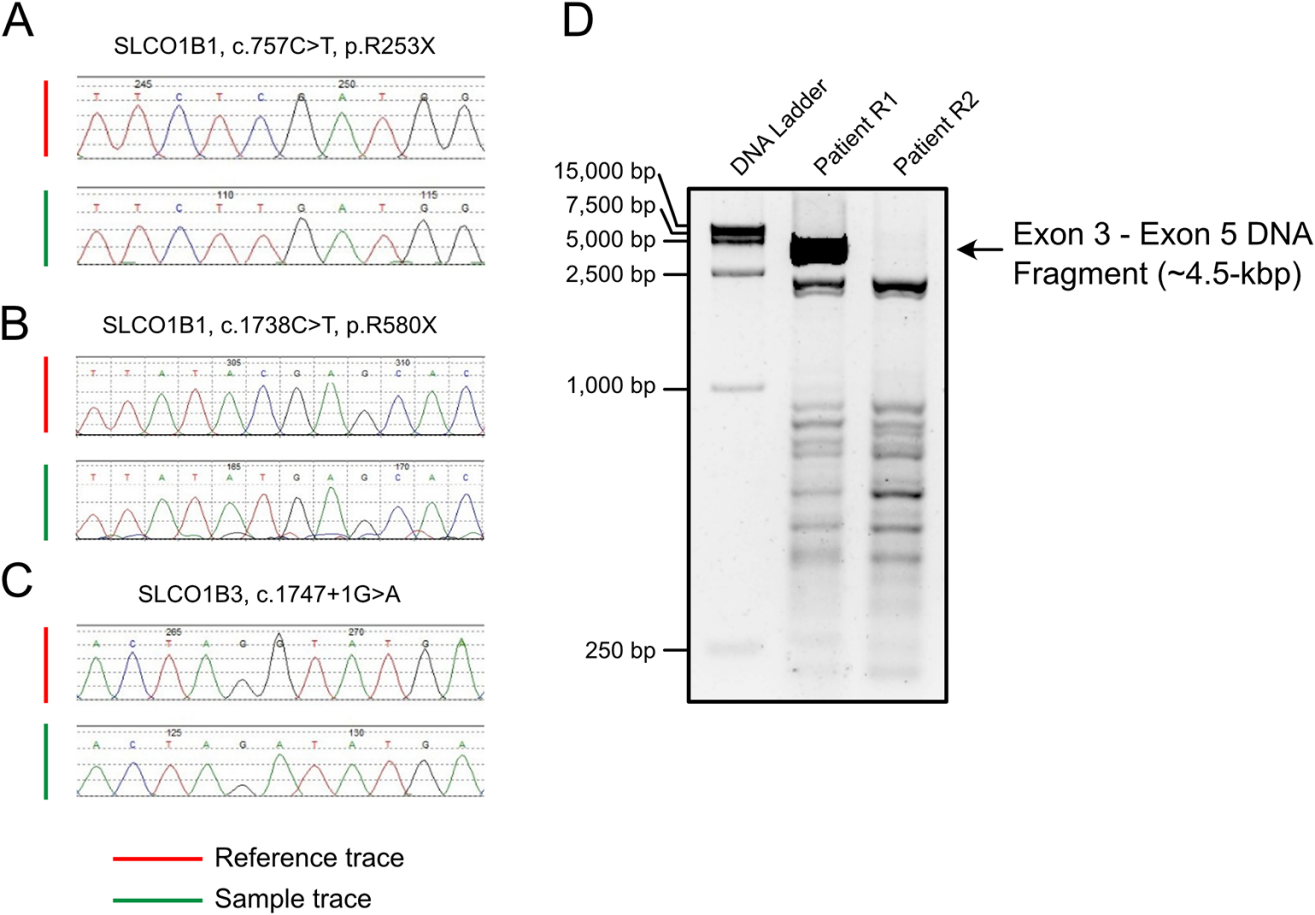
PCR amplification and Sanger sequencing of the target region in both patients with Rotor syndrome. **(A**–**C)** The sequences of three pathogenic point mutations. **(D)** Long-range PCR using the exon 3 forward primer and exon 5 reverse primer failed to produce the predicted ~4.5-kbp exon 3 to exon 5 amplification product.

All *SLCO1B1* and *SLCO1B3* exons, and at least 50 bp of flanking intronic sequences, were amplified from genomic DNA obtained from patients R1 and R2 using the primers listed in **Table S1**. All expected PCR products were obtained with the exception of *SLCO1B3* exon 4 in patient R2. Three pathogenic point mutations were identified in *SLCO1B1* and *SLCO1B3* following Sanger sequencing, including two nonsense mutations and one splicing mutation. Both patients were homozygous for all three genetic variants identified (**Figures 1A–C** and **Table 1**). In addition, three SNP sites were identified in *SLCO1B1* (**Table S2**). The clinical and predicted pathogenicity of the variants were integrated and obtained from VarCards.

**Table 1:** Pathogenic *SLCO1B1* and *SLCO1B3* variants identified in patients with Rotor syndrome.

Gene	Location	Mutation	Amino Acid Change	Patient	Mutation Type	Frequency (Asian/World)	Pathogenicity (ClinVar)	Prediction of Mutation Taster	Novel variants
SLCO1B1	Exon 8	c.757C > T	p.R253X	R1	Nonsense	0.0003/5.767e-05	Pathogenic	Disease Causing	
	Exon 13	c.1738C > T	p.R580X	R2	Nonsense	0.0047/0.0016	Pathogenic	Disease Causing	
SLCO1B3	Exon 4	NA	Premature termination codon	R2	Inversion	NA	NA	Disease Causing	Novel
	Intron 12	c.1747+1G > A	NA	R1	Splicing	NA	NA	Disease Causing	

### Inversion of *SLCO1B3* Exon 4 in Patient R2

**Figure 2:**
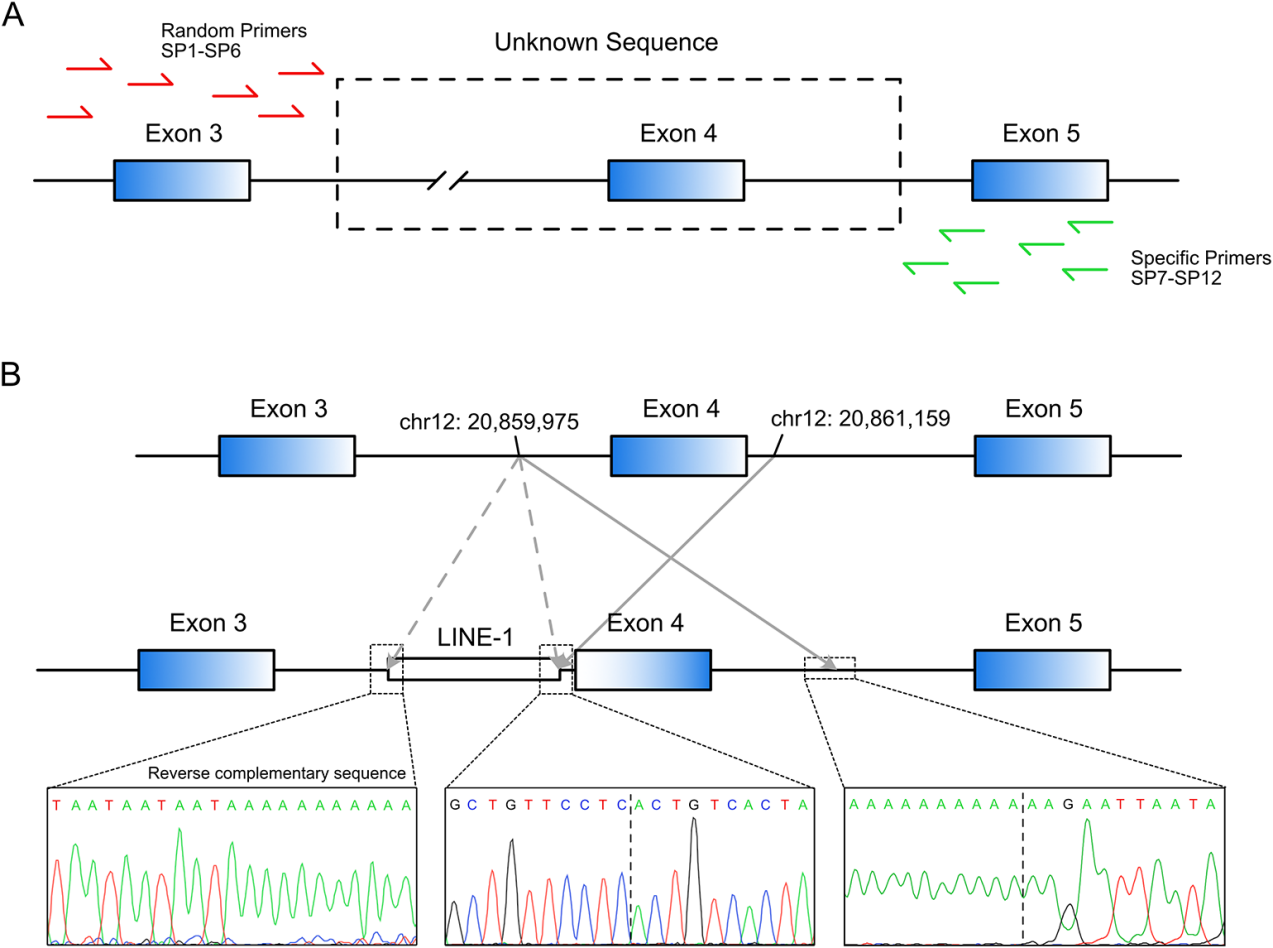
LINE-1-induced DNA inversion identified in *SLCO1B3*. **(A)** Genome walking analysis was used to identify the unknown sequence between *SLCO1B3* exons 3 and 5 in Patient R2. **(B)** The LINE-1 insertion reversed the fourth exon of *SLCO1B3*, 1,040 bp of 5′ sequence, and the 3′ GTAAGAATTAATAGTGACAGT, end-to-end in the homozygous state.

Long-range PCR using the forward primer for exon 3 and reverse primer for exon 5 failed to produce the predicted ~4.5-kbp exon 3–5 product in patient R2 (**Figure 1D**). We hypothesized that the missing target product may be the result of a DNA rearrangement around the fourth exon in *SLCO1B3*. To investigate this, genome walking analysis was performed (both forward and reverse) to identify the unknown sequences between exons 3 and 5. Specific primers were designed around exon 3 or 5 (**Figure 2A**). The amplification products were separated by agarose gel electrophoresis, extracted from the gel, and inserted into a simple T-vector for Sanger sequencing. Further genome walking analysis was then performed based on the newly identified DNA sequence. The results showed that *SLCO1B3* exon 4, 1,042 bp of 5′ sequence, and the 3′ GTAAGAATTAATAGTGACAGT (chr12: 20,859,975-20,861,159), were reversed end-to-end in the homozygous state (**Figure 2B** and **Supplemental File S1**).

### Inversion of the Fourth Exon Caused Aberrant Splicing and Generated Premature Stop Codon

**Figure 3:**
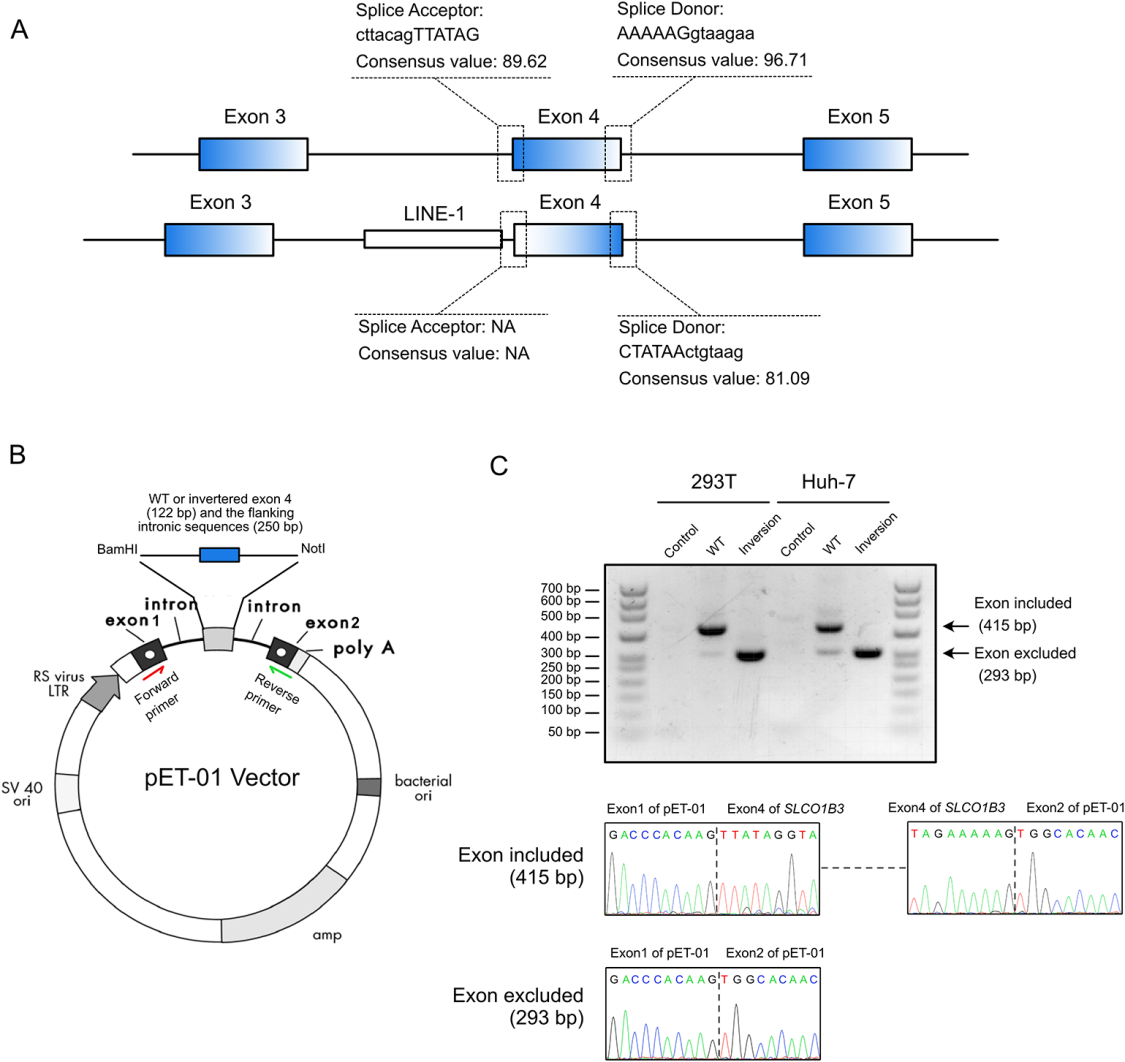
The inverted exon 4 was absent in the mature mRNA. **(A)** The splice acceptor and donor sites were destroyed after the inversion of exon 4. **(B)** The construction of pET-01 vector containing either the wild-type or inverted exon 4 of *SLCO1B3* (Reproduced from the handbook of pET-01 vector, MoBiTec, www.mobitec.com). **(C)** The splicing assay indicated the absent of the fourth exon of *SLCO1B3* in mature mRNA.

Splice site analysis indicated that the original splice acceptor and splice donor sites were destroyed after the inversion of exon 4 (**Figure 3A**), causing complete loss of exon 4 (122 bp) in the mature *SLCO1B3* mRNA. We constructed a splicing-reporter vector (pET-01) containing either the wild-type or inverted exon 4, and 250 bp of flanking intronic sequences (**Figure 3B**). RT-PCR showed that the inverted exon 4 was absent after the splicing of the pre-mRNA, both in 293T and Huh-7 cells. Thus, for *SLCO1B3* gene, a premature stop codon might generated (**Figure 3C** and **Supplemental File S2**).

### Exon 4 Inversion May Have Been Caused by the Insertion of LINE-1 in *SLCO1B3* Intron 3

Next, we investigated how the DNA inversion was induced. Further sequence analysis revealed that a reverse-complementary LINE-1 retrotransposon (~6 kbp) was inserted upstream of the inverted DNA fragment (**Figure 2B**). This was confirmed by long-range PCR (~10 kbp product) and Sanger sequencing (**Supplemental File S1**). LINE-1 was reported to be able to suppress gene transcription and induce exonization and exon skipping (Ostertag and Kazazian, 2001; Chen et al., 2006; Kalliokoski and Niemi, 2009). Therefore, we believe that the identified LINE-1 insertion caused the observed inversion of the entire exon 4 and the adjacent intronic sequence in the *SLCO1B3* gene of patient R2.

## Discussion

To date, only 26 and 10 pathogenic variants have been identified in *SLCO1B1* and *SLCO1B3*, respectively (based on the Human Gene Mutation Database, http://www.hgmd.cf.ac.uk/). Here, for the first time, we report *SLCO1B1* and *SLCO1B3* gene mutations in Chinese patients with Rotor syndrome. Unlike several other hereditary liver metabolic diseases, such as Wilson disease and hereditary hemochromatosis (Lv et al., 2016; Li et al., 2019), the hotspots for *SLCO1B1* and *SLCO1B3* mutations are quite similar between different populations. We identified one nonsense variant (*SLCO1B1*, c.757C > T, p.R253X) and one splicing variant (*SLCO1B3*, c.1747+1G > A) in patient R1, and one nonsense variant (*SLCO1B1*, c.1738C > T, p.R580X) and an exon 4 inversion in the *SLCO1B3* gene of patient R2. With the exception of the exon 4 inversion, these identified variants have been reported previously (Kagawa et al., 2015). Genome walking analysis showed that a 1,185 bp DNA segment, containing the entire exon 4 and adjacent intron sequence, was reversed end-to-end in *SLCO1B3*. The inversion was most likely induced by the insertion of a LINE-1 retrotransposon into *SLCO1B3* intron 3.

LINE-1 is the most abundant active autonomous transposon in mammalian genomes (Ostertag and Kazazian, 2001). Most of the recent research focused on the activity and regulation of LINE-1 in cancer and in nervous system, as well as being regulators of gene expression (Thomas et al., 2012; Elbarbary et al., 2016; Burns, 2017; Rodic, 2018; Saleh et al., 2019). Moreover, in earlier investigations, LINE-1 was reported to be able to affect the human genome, generating insertion mutations, altering gene expression, causing genomic instability, and finally, contributing to genetic innovation (Symer et al., 2002; Cordaux and Batzer, 2009). The intronic LINE-1 insertion can induce diseases through transcription suppression and promotion of exon inversion or skipping (Ostertag and Kazazian, 2001; Chen et al., 2006; Lee et al., 2008; Kalliokoski and Niemi, 2009). Similar cases have been previously reported for Rotor syndrome, a LINE-1 insertion was shown to result in the skipping of exon 5 or exons 5–7 and the introduction of stop codons in *SLCO1B3* transcripts from six Japanese patients (Kagawa et al., 2015).

Although no specific clinical treatment is required for Rotor syndrome, the absence or dysfunction of the OATP1B1 and OATP1B3 hepatic proteins may have serious consequences for the hepatic uptake and clearance of numerous commonly used drugs, including anticancer drugs and some angiotensin-converting enzyme inhibitors (Kalliokoski and Niemi, 2009; Niemi et al., 2011; Shitara, 2011). Thus, precise identification of *SLCO1B1* and *SLCO1B3* pathogenic variants by genetic analysis should be used to confirm the diagnosis of Rotor syndrome and to provide guidance for therapeutic drug prescriptions and genetic counseling.

## Data Availability Statement

The data supporting the conclusions of this article will be made available by the authors, without undue reservation, to any qualified researcher.

## Ethics Statement

The two patients provided written informed consent, and this study was approved by the Ethics Committee of Beijing Friendship Hospital, Capital Medical University.

## Author Contributions

JH and HY designed the study. DZ and SQ analyzed and interpreted the data and wrote the manuscript. SQ, AX, XL, BZ, YL, SJ, and HW performed the experiments. WZ and LW provided patients' samples and clinical data. JJ, XO, JH, and HY advised on the conception and supervised the study. JH revised the paper. All authors vouch for the data and analysis, approve the final version, and agree to publish the manuscript.

## Funding

This study was supported by grants from the Digestive Medical Coordinated Development Center of Beijing Municipal Administration of Hospitals (No. XXX0101, XXZ0501, XXT03), the National Key Technologies R&D Program (No. 2015BAI13B09), the National Natural Science Foundation of China (No. 81602032, 81800451), and the Beijing Postdoctoral Research Foundation (No. 2018-22-116).

## Conflict of Interest

The authors declare that this research was conducted in the absence of any commercial or financial relationships that could be construed as a potential conflict of interest.
