# Supplementary material for: Insertion of LINE-1 Retrotransposon Inducing Exon Inversion Causes a Rotor Syndrome Phenotype
Source: Front Genet. 2020 Jan 31;10:1399. doi: 10.3389/fgene.2019.01399 (PMC7005217; doi:10.3389/fgene.2019.01399)
Supplement: Supplementary file 1 [file DataSheet_1.docx]

**Supplemental File S1. The mutant sequence of *SLCO1B3* exon 3-5 in patient R2.**

【Intron 2】

......acatggtctttgagggaaggtacaatgtcttgggcatatttgcattcatttggggcattcagttctactagatacaaaattacactaagtcatatcaacataattttgttttttttctag

【Intron 2】

【Exon 3】

GAAATTTGCTTGTGATTGTATTTGTAAGTTACTTTGGATCTAAACTACACAGACCGAAGTTAATTGGAATTGGTTGTCTCCTTATGGGAACTGGAAGTATTTTGACATCTTTACCACATTTCTTCATGGGATA

【Exon 3】

【Intron 3】

gtaagtgttaaacagctctgagccatttattatcagctacttgtaaattagcagtagaattttatttttatacttgtaagtgggcagttaccttttgagaggaatacctataggtatgcatagagaaatggagtatatttcctatataatagttcatgtattgctttattcatcatgtatttctttatttatcatggcttttataaacttttaaataagaataatattatataaggtataCagttttatatgaaatgaatatcttatttaccaattttagtataacatatgtattttatgtttttgcctgtaagattttgattatcctcagtaaggcttcacaacttcacctgctgctgtacaggttttcccatcagcatttcactgaagctgctctttctaaggtaactgtgtagttatgattagagtaagaagctcatatgagaaaactgaaaatagcaataacttaaataagatagaagtttattttttacattttttcaagtccaaggatgatatgattctacttttcatcatagtgctatgccttgcatggccttgtcctcttgttacaaattgtggcaatgtatttccaggcaacaggatagaagaagagaagaacaagaacaaagagtgcacacaggctgagctccaggagcattttcagaagcaaaacactttcactgatttcacatttacgggaagtcagttacgttttacacatagatatgtagatgttggaaaaatgtactttattccaggcttccgtatttccagctaaaaaaaccaacttctgttactatggaaggagagaagaaaggatactgagagacaacgagcAgtttgttagagacaccaattccttaatgccaaatccagcaatcatgcctgttggtcaccatatgaattctgtttttttccccctctacatttggcccttataagaactttcttcttaatggtactgctctaatactcatcatattccacaatcttgattcatttttctatttttgtggcttcatcatgcccatctttttcctttgcttctgacaccataatttctgtgtcccataattccctctaatcttcttaatatcttattcttcattatttttatggagaatatgccttaaatgcaactaccccagcttcatcttccattgctccccacatttgaggaccacattctaggcttacagttctctaatgtgccataatatgtcatatctttgcatctgttatttctacagacttgaatacttttcttcctttgtctaccaatttgaattctacccatcttcaatattcatttaatctgtccttcacattgtgaagttttctttgatctaccctcacctccaagaaaagctgatcatttctttttttttttttttttttttNNNNNNNNNN

【Intron 3】

【Inserted LINE-1】

NNNNNNNNNNNNttttttttattattattattattattattatttttttttattatactctaagttttagggtacatgtgcacattgtgcaggttagttacatatgtatacatgtgccatgctggtgcgctgcacccactaatgtgtcatctagcattaggtatatctcccaatgctatccctcccccctcccccgaccccaccacagtccccagagtgtgatattccccttcctgtgtccatgtgatctcattgttcaattcccacctatgagtgagaatatgcggtgtttggttttttgttcttgcgatagtttactgagaatgatggtttccaatttcatccatgtccctacaaaggatatgaactcatcattttttatggctgcatagtattccatggtgtatatgtgccacattttcttaatccagtctatcattgttggacatttgggttggttccaagtctttgctattgtgaatagtgccgcaataaacatacgtgtgcatgtgtctttatagcagcatgatttatactcatttgggtatatacccagtaatgggatggctgggtcaaatggtatttctagttctagatccctgaggaatcgccacactgacttccacaatggttgaactagtttacagtcccaccaacagtgtaaaagtgttcctatttctccgcatcctctccagcacctgttgtttcctgactttttaatgattgccattctaactggtgtgagatgatatctcatagtggttttgatttgcatttctctgatggccagtgatgatgagcatttcttcatgtgttttttggctgcataaatgtcttcttttgagaagtgtctgttcatgtccttcgcccactttttgatggggttgtttgtttttttcttgtaaatttgtttgagttcattgtagattctggatattagccctttgtcagatgagtaggttgcaaaaattttctcccatgttgtaggttgcctgttcactctgatggtagtttcttttgctgtgcagaagctctttagtttaattagatcccatttgtcaattttgtcttttgttgccattgcttttggtgttttggacatgaagtccttgcccacgcctatgtcctgaatggtaatgcctaggttttcttctagggtttttatggttttaggtttaacgtttaaatctttaatccatcttgaattgatttttgtataaggtgtaaggaagggatccagtttcagctttctacatatggctagccagttttcccagcaccatttattaaatagggaatcctttccccattgcttgtttttctcaggtttgtcaaagatcagatagttgtagatatgcggcattatttctgagggctctgttctgttccattgatctatatctctgttttggtaccagtaccatgctgttttggttaccgtagccttgtagtatagtttgaagtcaggtagtgtgatgcctccagctttgttcttttggcttaggattgacttggcaatgcgggctcttttttggttccatatgaactttaaagtagttttttccaattctgtgaagaaagtcattggtagcttgatggggatggcattgaatctgtaaattaccttgggcagtatggccattttcacgatattgattcttcctacccatgagcatggaatgttcttccatttgtttgtctcctcttttatttccttgagcagtggtttgtagttctccttgaagaggtccttcacatcccttgtaagttggattcctaggtattttattctctttgaagcaattgtgaatgggagttcacccatgatctggctctctgtttgtctgttgttggtgtataagaatgcttgtgatttttgtacattgattttgtatcctgagactttgctgaagttgcttatcagcttaaggagattttgggctgagacgatggggttttctagataaacaatcatgtcgtctgcaaacagggacaatttgacttcctcttttcctaattgaataccctttatttccttctcctgcctgattgccctggccagaacttccaacactatgttgaataggagcggtgagagagggcatccctgtcttgtgccggttttcaaagggaatgcttccagtttttgcccattcagtatgatattggctgtgggtttgtcatagatagctcttattattttgaaatacgtcccatcaatacctaatttattgagagtttttagcatgaagggttgttgaattttgtcaaaggctttttctgcatctattgagataatcatgtggtttttgtctttggctctgtttatatgctggattacatttattgatttgcgtatattgaaccagccttgcatcccagggatgaagcccacttgatcatggtggataagctttttgatgtgctgctggattcggtttgccagtattttattgaggatttttgcatcaatgttcatcaaggatattggtctaaaattctcttttttggttgtgtctctgcccggctttggtatcagaatgatgctggcctcataaaatgagttagggaggattccctctttttctattgattggaatagtttcagaaggaatggtaccagttcctccatgtacctctggtagaattcagctgtgaatccatctggtcctggactctttttggttggtaaactattgattattgccacaatttcagagcctgttattggtctattcagagattcaacttcttcctggtttagtcttgggagagtgtatgtgtcgaggaatgtatccatttcttctagattttctagtttatttgcgtagaggtgtttgtagtattctctgatggtagtttgtatttctgtgggatcggtggtgatatcccctttatcattttttattgtgtctatttgattcttctctctctttttctttattagtcttgctagcggtctatcaattttgttgatcctttcaaaaaaccagctcctggattcattaattttttgaagggttttttgtgtctctatttccttcagttctgctctgattttagttatttcttgccttctgctagcttttgaatgtgtttgctcttgcttttctagttcttttaattgtgatgttagggtgtcaattttggatctttcctgctttctcttgtaggcatttagtgctataaatttccctctacacactgctttgaatgcgtcccagagattctggtatgtggtgtctttgttctcgttggtttcaaagaacatctttatttctgccttcatttcgttatgtacccagtagtcattcaggagcaggttgttcagtttccatgtagttgagcagctttgagtgagattcttaatcctgagttctagtttgattgcactgtggtctgagagatagtttgttataatttctgttcttttacatttgctgaggagagctttacttccaactatgtggtcaattttggaataggtgtggtgtggtgctgaaaaaaatgtatattctgttgatttggggtggagagttctgtagatgtctattaggtctgcttggtgcagagctgagttcaattcctgggtatccttgttgactttctgtctcgttgatctgtctaatgttgacagtggggtgttaaagtctcccattattaatgtgtgggagtctaagtctctttgtaggtcactgaggacttgctttatgaatctgggtgctcctgtattgggtgcataaatatttaggatagttagctcctcttgttgaattgatccctttaccattatgtaatggccttctttgtctcttttgatctttgttggtttaaagtctgttttatcagagactaggattgcaacccctgcctttttttgttttccattggcttggtagatcttcctccatccttttattttgagcctatgtgtgtctctgcacgtgagatgggtttcctgaatacagcacactgatgggtcttgactctttatccaacttgccagtctgtgtcttttaattgcagaatttagtccatttatatttaaagttaatattgttatgtgtgaatttgatcctgtcattatgatgttagctggtgattttgctcattagttgatgcagtttcttcctagtctcgatggtctttacattttggcatgattttgcagcggctggtaccggttgttcctttccatgtttagcgcttccttcaggagctcttttagggcaggcctggtggtgacaaaatctctcaacatttgcttgtctataaagtattttatttctccttcacttatgaagcttagtttggctggatatgaaattctgggttgaaaattcttttctttaagaatgttgaatattggcccccactctcttctggcttgtagggtttctgccgagagatccgctgttagtctgatgggctttcctttgagggtaacccgacctttctctctggctgcccttaacattttttccttcatttcaactttggtgaatctgacaattatgtgtcttggagttgctcttctcgaggagtatctttgtggcgttctctgtatttcctgaatctgaacgttggcctgccttgctagattggggaagttctcctggataatatcctgcagagtgttttccaacttggttccattctccacatcactttcaggtacaccaatcagacgtagatttggtcttttcacatagtcccatatttcttggaggctttgctcatttctttttattcttttttctctaaacttcccttctcgcttcatttcattcatttcatcttccattgctgataccctttcttccagttgatcgcatcggctcctgaggcttctgcattcttcacgtagttctcgagccttggttttcagctccatcagctcctttaagcacttctctgtattggttattctagttatacattcttctaaatttttttcaaagttttcaacttctttgcctttggtttgaatgtcctcccgtagctcagagtaatttgatcgtctgaagccttcttctctcagctcgtcaaaatcattctccatccagctctgttccgttgctggtgaggaactgcgttcctttggaggaggagaggcgctctgcgttttagagtttccagtttttctgttctgttttttccccatctttgtggttttatctacttttggtctttgatgatggtgatgtacagatgggttttcggtgtagatgtcctttcttgttgttagttttccttctaacagacaggaccctcagctgcaggtctgttggaataccctgccgtgtgaggtgtcagtgtgcccctgctggggggtgcctcccagttaggctgctcgggggtcaggagtcagggacccacttgaggaggcagtctgcccgttctcagatctccagctgcgtcctgggagaaccactgctctcttcaaagctgtcagacagggacacttaagtctgcagaggttactgctgtctttttgtttgtctgtgccctgcccccagaggtggagcctacagaggcaggcaggcctccttgagctgtggtgggctccacccagttcgagcttcccggctgctttgtttacctaagcaagcctgggcaatggcgggcgcccctcccccagcctcgctgccgccttgcagtttgatctcagactgctgtgctagcaatcagcgagattccgtgggcgtaggaccctccgagccaggtgtgggatatagtctcgtggtgcgccgtttcttaagccggtctgaaaagcgcaatattcgggtgggagtgacccgattttccaggtgcgtccgtcacccctttctttgactcggaaagggaactccctgaccccttgcgcttcccaggtgaggcaatgcctcgccctgcttcggctcgcgcacggtgcgcacacacactggcctgcgcccactgtctggcactccctagtgagatgaacccggtacctcagatggaaatgcagaaatcaccgtcttctgcgtcgctcacgctgggagctgtagaccggagctgttcctc

【Inserted LINE-1】

【Inverted Exon 4 and flanking intronic sequences】

actgtcactattaattcttacCTTTTTCTACTATCTCAGGTGATGTTCCATTGAATGATAAGGTTTGATTAATTAAACAGGTTGATAAACTTGATGTTGAATTTTCTGATGGATTAATATGGGTTTCTTTAGAATACCTATAActgtaagagcaacatgaaattgaacattttgcttatctactgaatattttcagttttcccagaaatacagagaaattttctcctttatttgaaccgctgtcttctccaaattaccagaatcttgtttgtcaacttcccagcatgtatttcagaacaggtgatgagagggggtgaaatgggagtgtccactaagaaagatacttcaatacaccatgaacaaagtgggttatcaccttgcttttatcctaagagtgcttataatctggtcaagatatgaaatatcatggtttcacctgttgtttttttatctgttaactatgtctatttatccctttgagttaccatcaaagatggtcaatctgtctgattttctttcatgtacacacacacacacacacacacacacacacacacaaagtgcttgacttttcaaaactcaataaaaacaaatttctaattcatttaaactttatgaggaaaaaataagaacaacggagatggagctggatcagggacagacgcTgatgctcaaaaatactaaccgctctgtgagttctaaggacaacatatagtgtgccaggcattttgctcaacttcaaagaacaagaaataaaacatgaagatcaagaaataaaacacacagtgtttgtactttggaaatttcacactctctaagataaatgtgtatgatcgaagatatagtaaatttaatatacaaggtcctgggttgtcacacacccacaaaaaagaaatgatcaggggctgggcgcggtggctcacgcctgtaatcccagcactttgggaggtggaggcgggcagatcacgaggtcaggagatccagaccaccctggctaacacagtgaaaccccgtctctactaaaaatacaaaaaattagccgggcgtggtggcaggcgcctgtagtcccagctactcgggaggctgaggcaggagaatggtgtgaacccgggaggcggggcttgcagtgagccgagatcgcgccactgcactccagcctgggcgactagcgagactccgtctc

【Inverted Exon 4 and flanking intronic sequences】

【Inserted sequences】

aaaaaaaaaaaaaaaaaa

【Inserted sequences】

【Intron 4】

aagaattaatagtgacagtaaaacaaattctagattgatagatttaatcacgtctataaagtttctgatattctttaacaaaattgatttaagaacaaataggaagaacatttatcccttacatacagacaaaatcatactgttaatgtatgcagtcgattcttaaacaacacaggtttgaactgcacctgttcgcttatatgcagcttttgtccaaccaaacagaaggagaaacccaccaccgatgtggaggtctgacttctataggcggtttccacagggcaaatgagaggacttaagtctgtataaatttgtgtacatatgagttgtcctagaacaaacctcctagtgtacaaatggattattgtataccaagggcatttagaagtagataagaaaaaagaacaaatttagtaggaaatttgcaaagaacatacacatttaaaataaaaaagcagaaagtggtagaaatatgtgaataatataactttctctttataaagatgcaaaatgttatagcatttgaagactttgtaactattataaacacaataatataagtataattttaaccccaaaatttattaaaaataaatgaagtggaggaaaaaaaatgatttcaagttttctgtattgctgggagagtataaatttcaaagtctttctggagattaattttcaatacatattaaagaacaaaaatatacttgcctactttcttgattaatcttaaggaaatcatcagtaatgttcacaaggacatttcataatcacagccatgacatccatgtttaacagaataaaaaaaaatattttgtaacgacaaaagaagagattgtttaaccaaattaggaaactattattcaacacactactatgcagtaattaaaattttaatatagaagaactcttgacaacatggggaaattgtgacaatatagtgataattgtatatgcaccttaaaaataacctggatttttaaatatgtaatgtacataaggaatattatgcatattttgtgcactaaaaatgcagaagatctagaataacatttgcaatgatcgtctgtgtgtagtgtaattatagaaaatttttacttgttttctttgtgcccttccttattttcaaactttgtaaagtgaatatgaatcacttgtaattaggaaacaaacaaacaaaaaatggaaaaatgaaggatttaaagtagttaaatttctaataggaatgttaaaattaatgtttaaagtaaaacactctcttgtctcgatag

【Intron 4】

【Exon 5】

ATTGTGTAAAGGAATCTGGGTCACACATGTGGATCTATGTCTTCATGGGGAATATGCTTCGTGGCATAGGGGAAACCCCCATAGTACCATTGGGGATTTCATACATTGATGATTTTGCAAAAGAAGGACATTCTTCCTTGTATTTAG

【Exon 5】

【Intron 5】

gtaacgtacagaatatattaaatttctgatatacattccctggatctacccttga......

【Intron 5】
