# Supplementary material for: Insertion of LINE-1 Retrotransposon Inducing Exon Inversion Causes a Rotor Syndrome Phenotype
Source: Front Genet. 2020 Jan 31;10:1399. doi: 10.3389/fgene.2019.01399 (PMC7005217; doi:10.3389/fgene.2019.01399)
Supplement: Supplementary file 2 [file DataSheet_2.docx]

**Supplemental File S2. The predicted protein sequence of *SLCO1B3* gene with inverted exon 4.**

SLCO1B3_WT MDQHQHLNKTAESASSEKKKTRRCNGFKMFLAALSFSYIA 40

SLCO1B3_Inversion MDQHQHLNKTAESASSEKKKTRRCNGFKMFLAALSFSYIA 40

SLCO1B3_WT KALGGIIMKISITQIERRFDISSSLAGLIDGSFEIGNLLV 80

SLCO1B3_Inversion KALGGIIMKISITQIERRFDISSSLAGLIDGSFEIGNLLV 80

SLCO1B3_WT IVFVSYFGSKLHRPKLIGIGCLLMGTGSILTSLPHFFMGY 120

SLCO1B3_Inversion IVFVSYFGSKLHRPKLIGIGCLLMGTGSILTSLPHFFMG* 120

SLCO1B3_WT YRYSKETHINPSENSTSSLSTCLINQTLSFNGTSPEIVEK 160

SLCO1B3_Inversion LCKGIWVTHVDLCLHGEYASWHRGNPHSTIGDFIH**FCK 160

SLCO1B3_WT DCVKESGSHMWIYVFMGNMLRGIGETPIVPLGISYIDDFA 200

SLCO1B3_Inversion RRTFFLVF-------------------------------- 168

SLCO1B3_WT KEGHSSLYL 209

SLCO1B3_Inversion --------- 168
