# Supplementary material for: Insertion of LINE-1 Retrotransposon Inducing Exon Inversion Causes a Rotor Syndrome Phenotype
Source: Front Genet. 2020 Jan 31;10:1399. doi: 10.3389/fgene.2019.01399 (PMC7005217; doi:10.3389/fgene.2019.01399)
Supplement: Supplementary file 3 [file Table_1.docx]

| Table S1. Primers used for sequence analysis of *SLCO1B1* and *SLCO1B3*. | | | | | | |
| --- | --- | --- | --- | --- | --- | --- |
| **Primers for *SLCO1B1* gene** | | | | | | |
| **Exon** | **Primer Sequence** | |  | **Exon** | **Primer Sequence** | |
| 1 | 5'-TGGCAACTGGAGTGAACTCTT-3' | |  | 9 | 5'-AGCTGTGAACAGCCTGTGGT-3' | |
|  | 5'-GGCTCTTCTACTCCCAGAAGG-3' | |  |  | 5'-TTTCACAATAAAATACCACTTGGA-3' | |
| 2 | 5'-TGATGATGATCTTGTGGCTTTT-3' | |  | 10 | 5'-GCACATTCTGCACATGTATCC-3' | |
|  | 5'-AAAAGACTGGTTTAAATCAAGGTATGA-3' | |  |  | 5'-GGAATAAAAGAATGTGTTTGAGGTC-3' | |
| 3 | 5'-ACCTGGTAAAAGGGAAAACTAAG-3' | |  | 11 | 5'-CCTTCTCCTCCCCTTCTTTG-3' | |
|  | 5'-TATTGCCAAATTGCCTGTGA-3' | |  |  | 5'-CATCACACCCATCACAATAACA-3' | |
| 4 | 5'-TGTCTTTGAGGGAAGGCACT-3' | |  | 12 | 5'-AATGCAATGTATTTGCAGCAC-3' | |
|  | 5'-TGGGTATCTTCTCAAAAGGTAACTG-3' | |  |  | 5'-CAGCCTTGAGAGTTCATAGTAATTTT-3' | |
| 5 | 5'-GGGGAAGATAATGGTGCAAA-3' | |  | 13 | 5'-TAATGGGGCCATTCAACTGT-3' | |
|  | 5'-TGTTGTTAATGGGCGAACTG-3' | |  |  | 5'-TTTATTTATCACAGAAATAGAAAGGA-3' | |
| 6 | 5'-TGTCAAAGTTTGCAAAGTGAA-3' | |  | 14 | 5'-TTGGGTAGATGCAGAACAAAA-3' | |
|  | 5'-TTCAAAAGTAGACAAAGGGAAAGTG-3' | |  |  | 5'-CCATACTGCATGCAAAGTCAG-3' | |
| 7 | 5'-CCATGCATTCTTGGCATCTA-3' | |  | 15 | 5'-TCGTTATGCCCCAATAAAAA-3' | |
|  | 5'-GGAGCTGGATTTTATATTTATTCTGA-3' | |  |  | 5'-ATCAATGCAATGCTGTTTGG-3' | |
| 8 | 5'-TCGTGTCTTGGAATTGAGGA-3' | |  |  |  | |
|  | 5'-GCAAAAGAAAGCCAACTCCA-3' | |  |  |  | |
|  | | | | | | |
| **Primers for *SLCO1B3* gene** | | | | | | |
| **Exon** | **Primer Sequence** | |  | **Exon** | **Primer Sequence** | |
| 1 | 5'-TTGAGCTTGTGGCTTTTCCT-3' | |  | 8 | 5'-GCGACTCTCTTAGAAAGCCTCA-3' | |
|  | 5'-ACGCTTCAATGGAAAAATTG-3' | |  |  | 5'-TCAAATGCAGAACAACGATGA-3' | |
| 2 | 5'-TTTCAACTTGTATAGGGAAAAATGG-3' | |  | 9 | 5'-CCAGGTTCTCCACCCTTCTC-3' | |
|  | 5'-CAAATTGCCCATGAGAGACA-3' | |  |  | 5'-GGAAATGGACATTTTAAGAATTTGA-3' | |
| 3 | 5'-ACATGGTCTTTGAGGGAAGG-3' | |  | 10 | 5'-TCCCCTTGTCTCCCTCTTCT-3' | |
|  | 5'-TCTCAAAAGGTAACTGCCCACT-3' | |  |  | 5'-TTCACAGCTATCACGAGGACA-3' | |
| 4 | 5'-GTGGACACTCCCATTTCACC-3' | |  | 11 | 5'-CAGGGAGCTATTTTGCCTTC-3' | |
|  | 5'-GGTGCAGTTCAAACCTGTGTT-3' | |  |  | 5'-TGCCTATCATTAGGTGTGTTTTAGTC-3' | |
| 5 | 5'-TGTTTTCTTTGTGCCCTTCC-3' | |  | 12 | 5'-AGGGAGAGGAATGATGCTGA-3' | |
|  | 5'-TCAAGGGTAGATCCAGGGAAT-3' | |  |  | 5'-CACTCAATTTTCCCGTTCCT-3' | |
| 6 | 5'-TTCCCTGGATCTACCCTTGA-3' | |  | 13 | 5'-TTTGATTCCTGGGTGGATGT-3' | |
|  | 5'-GGAAGAATGGTGTCCTGCAC-3' | |  |  | 5'-AAAATGAGATACCAGAATGCTTGA-3' | |
| 7 | 5'-CAATTGCAAGATGTCATCAACC-3' | |  | 14 | 5'-ATCTTATGCCCCCAATGAAA-3' | |
|  | 5'-CTCACTTCTCCGTTTCATTGAG-3' | |  |  | 5'-CCATGGGTACTCCCATTTTT-3' | |
|  | | | | | | |
| **Primers for genome walking analysis** | | | | | | |
| SP1 | 5'- TCCTCCAGGAAACTTTCACAAAGC-3' | |  | SP7 | 5'- TAGCAAACAGAGATCCCAGTGCAA-3' | |
| SP2 | 5'- CATGGTCTTTGAGGGAAGGTACAA-3' | |  | SP8 | 5'- TTTCAAGGGTAGATCCAGGGAATG-3' | |
| SP3 | 5'- GTGGGCAGTTACCTTTTGAGAGGA-3' | |  | SP9 | 5'- GATCCACATGTGTGACCCAGATTC-3' | |
| SP4 | 5'- CTCCAGGAGCATTTTCAGAAGCAA-3' | |  | SP10 | 5'- ACACTACACACAGACGATCATTGC-3' | |
| SP5 | 5'- CAGGCTTCCGTATTTCCAGCTAAA-3' | |  | SP11 | 5'- CCCCATGTTGTCAAGAGTTCTTCT-3' | |
| SP6 | 5'- CATGCCTGTTGGTCACCATATGAA-3' | |  | SP12 | 5'- AAACATGGATGTCATGGCTGTG-3' | |
|  | | | | | | |
| **Primers for splicing assay** | | | | | | |
| pET-Fw | | 5'-GATCGATCCGCTTCCTGCCC-3' |  | pET-Rv | | 5'-GTAGAGAGAGCAGATGCTGGTG-3' |
