# Supplementary material for: Insertion of LINE-1 Retrotransposon Inducing Exon Inversion Causes a Rotor Syndrome Phenotype
Source: Front Genet. 2020 Jan 31;10:1399. doi: 10.3389/fgene.2019.01399 (PMC7005217; doi:10.3389/fgene.2019.01399)
Supplement: Supplementary file 4 [file Table_2.docx]

| Table S2. SNP sites identified in patients with Rotor syndrome. | | | | | | | | | | | | | |
| --- | --- | --- | --- | --- | --- | --- | --- | --- | --- | --- | --- | --- | --- |
| **Gene** | **Location** | **Mutation** | **Amino Acid Change** | **Patient** | **Mutation**  **Type** | **Frequency (Asian/World)** | **Pathogenicity（ClinVar）** | **Prediction of pathogenicity (Scores)** | | | | |  |
|  |  |  |  |  |  |  |  | **Polyphen-2** |  | **SIFT** |  | **Mutation Taster** |  |
| *SLCO1B1* | Exon 5 | c.388A>G | p.N130D | R1 | Missense | 0.7499 /0.4795 | Benign | Benign |  | Tolerable |  | Polymorphism |  |
|  | Exon 6 | c.571T>C | p.L191L | R2 | synonymous | 0.2497/0.5260 | Benign | NA |  | NA |  | NA |  |
|  | Exon 6 | c.597C>T | p.F199F | R1 | synonymous | 0.5109/0.3851 | Benign | NA |  | NA |  | NA |  |
